# Supplementary material for: Elemental and Speciation Analyses of Different Brands of Yerba Mate (Ilex paraguariensis)
Source: Foods. 2021 Nov 26;10(12):2925. doi: 10.3390/foods10122925 (PMC8700693; doi:10.3390/foods10122925)
Supplement: Supplementary file 1 [file foods-10-02925-s001.zip › foods-1460075-supplementary.pdf]

Supplementary Material

Elemental and Speciation Analyses of Different Brands of Yerba Mate

(Ilex Paraguariensis)

Jędrzej Proch, Aleksandra Orłowska, Przemysław Niedzielski*

Adam Mickiewicz University, Faculty of Chemistry, Department of Analytical Chemistry, Uniwersytetu Poznańskiego 8, 61–614 Poznań, Poland.

***Corresponding author**

mail: pnied@amu.edu.pl

Standard addition method

Table S1. Results of spiked recovery in standard addition method (two sample solutions, P1 and P2, obtained in ultrasound–assisted extraction were spiked with two concentrations of mixed standard solution).

		**P1**							**P2**						
**Analyte**	**QL** [mg L^{-1}]	**Sample** [mg L^{-1}]	**Added** [mg L^{-1}]	**Found** [mg L^{-1}]	**Recovery** [%]	**Added** [mg L^{-1}]	**Found** [mg L^{-1}]	**Recovery** [%]	**Sample** [mg L^{-1}]	**Added** [mg L^{-1}]	**Found** [mg L^{-1}]	**Recovery** [%]	**Added** [mg L^{-1}]	**Found** [mg L^{-1}]	**Recovery** [%]
**Al**[a]	0.009	0.432	2.00	2.396	98	10.0	10.51	101	0.603	2.00	2.617	109	10.0	10.57	101
**As**[a]	0.018	BQL	0.10	0.092	92	0.50	0.487	97	BQL	0.10	0.109	109	0.50	0.492	98
**As(III)**[b]	0.006	BQL	0.10	0.090	90	0.50	0.503	101	BQL	0.10	0.098	98	0.50	0.515	103
**As(V)**[b]	0.021	BQL	0.10	0.094	94	0.50	0.497	99	BQL	0.10	0.101	101	0.50	0.490	98
**DMA**[b]	0.019	BQL	0.10	0.089	89	0.50	0.486	97	BQL	0.10	0.085	85	0.50	0.471	94
**Cd**[a]	0.001	BQL	0.50	0.476	95	2.50	2.511	100	BQL	0.50	0.465	93	2.50	2.586	103
**Co**[a]	0.001	BQL	1.00	1.053	105	5.00	4.917	98	BQL	1.00	1.124	112	5.00	5.133	103
**Cu**[a]	0.001	0.032	1.00	0.980	95	5.00	5.148	102	0.071	1.00	1.073	104	5.00	4.967	99
**Fe**[a]	0.007	0.487	2.00	2.672	109	10.0	10.73	102	0.365	2.00	2.459	99	10.0	10.57	101
**Fe(II)**[c]	0.036	BQL	0.20	0.171	86	1.00	0.934	93	BQL	0.20	0.182	91	1.00	0.887	89
**Fe(III)**[c]	0.030	BQL	0.20	0.183	91	1.00	0.966	97	BQL	0.20	0.204	102	1.00	0.929	93
**Hg**[a]	0.004	BQL	0.50	0.528	106	2.50	2.523	101	BQL	0.50	0.476	95	2.50	2.667	107
**Li**[a]	<0.001	BQL	0.50	0.483	97	2.50	2.386	95	BQL	0.50	0.495	99	2.50	2.705	108
**Mn**[a]	0.006	4.568	5.00	9.771	104	25.0	31.70	109	5.209	5.00	10.18	112	25.0	30.48	104
**Mo**[a]	0.002	BQL	0.50	0.492	98	2.50	2.290	92	BQL	0.50	0.483	97	2.50	2.453	98
**Ni**[a]	0.005	0.032	0.50	0.534	100	2.50	2.466	97	0.031	0.50	0.491	92	2.50	2.519	99
**Pb**[a]	0.012	BQL	0.50	0.463	93	2.50	2.564	103	BQL	0.50	0.523	105	2.50	2.855	114
**Sb**[a]	0.017	BQL	0.10	0.085	85	0.50	0.470	94	BQL	0.10	0.103	103	0.50	0.508	102
**Se**[a]	0.007	BQL	0.10	0.115	115	0.50	0.521	104	BQL	0.10	0.096	96	0.50	0.550	110
**Zn**[a]	0.002	0.557	2.00	2.304	87	10.0	10.92	104	0.397	2.00	2.392	92	10.0	10.75	102

[a] – determined by ICP OES; [b] – determined by HPLC–HG–ICP OES; [c] – determined by HPLC–ICP OES; **QL** – instrument quantification limit (as 10 standard deviation of the blank) [mg L^{-1}]; **BQL** – below instrument quantification limit

Table S2. Results of spiked recovery in standard addition method (two sample solutions, P1 and P2, obtained in microwave–assisted digestion were spiked with two concentrations of mixed standard solution).

		P1							P2						
**Analyte**	**QL** [mg L^{-1}]	**Sample** [mg L^{-1}]	**Added** [mg L^{-1}]	**Found** [mg L^{-1}]	**Recovery** [%]	**Added** [mg L^{-1}]	**Found** [mg L^{-1}]	**Recovery** [%]	**Sample** [mg L^{-1}]	**Added** [mg L^{-1}]	**Found** [mg L^{-1}]	**Recovery** [%]	**Added** [mg L^{-1}]	**Found** [mg L^{-1}]	**Recovery** [%]
**Al**	0.009	0.892	2.00	2.821	96	10.0	9.206	83	0.690	2.00	2.899	110	10.0	9.089	84
**As**	0.018	BQL	0.10	0.085	85	0.50	0.420	84	BQL	0.10	0.089	89	0.50	0.418	84
**Cd**	0.001	0.004	0.50	0.478	95	2.50	2.473	99	0.003	0.50	0.475	94	2.50	2.344	94
**Co**	0.001	0.003	1.00	0.899	90	5.00	4.427	88	0.002	1.00	0.911	91	5.00	4.263	85
**Cr**	0.001	0.005	0.50	0.466	92	2.50	2.277	91	0.002	0.50	0.474	94	2.50	2.192	88
**Cu**	0.001	0.091	1.00	0.939	85	5.00	4.424	87	0.031	1.00	0.832	80	5.00	4.301	85
**Fe**	0.007	1.025	2.00	2.720	85	10.0	10.38	94	1.604	2.00	3.269	83	10.0	10.40	88
**Hg**	0.004	BQL	0.50	0.488	98	2.50	2.917	117	BQL	0.50	0.490	98	2.50	2.542	102
**Li**	<0.001	0.002	0.50	0.418	83	2.50	2.028	81	0.003	0.50	0.424	84	2.50	2.026	81
**Mn**	0.006	4.535	5.00	10.40	117	25.0	31.17	107	5.309	5.00	10.56	105	25.0	27.60	89
**Mo**	0.002	BQL	0.50	0.424	85	2.50	2.070	83	BQL	0.50	0.425	85	2.50	2.010	80
**Ni**	0.005	0.058	0.50	0.538	96	2.50	2.313	90	0.069	0.50	0.513	89	2.50	2.229	86
**Pb**	0.012	BQL	0.50	0.493	99	2.50	2.180	87	BQL	0.50	0.471	94	2.50	2.094	84
**Sb**	0.017	BQL	0.10	0.089	89	0.50	0.419	84	BQL	0.10	0.087	87	0.50	0.417	83
**Se**	0.007	BQL	0.10	0.088	88	0.50	0.443	89	BQL	0.10	0.086	86	0.50	0.437	87
**Zn**	0.002	0.422	2.00	2.099	84	10.0	9.546	91	0.383	2.00	2.123	87	10.0	9.238	89

QL – instrument quantification limit (as 10 standard deviation of the blank) [mg L^{-1}]; **BQL** – below instrument quantification limit

		Total																Extractable															
		**Al**	**As**	**Cd**	**Co**	**Cr**	**Cu**	**Fe**	**Hg**	**Li**	**Mn**	**Mo**	**Ni**	**Pb**	**Sb**	**Se**	**Zn**	**Al**	**As**	**Cd**	**Co**	**Cu**	**Fe**	**Hg**	**Li**	**Mn**	**Mo**	**Ni**	**Pb**	**Sb**	**Se**	**Zn**
**Total**	**Al**	1.00																														
	**As**	0.21	1.00																													
	**Cd**	0.05	−0.01	1.00																												
	**Co**	**−0.45**	−0.22	−0.12	1.00																											
	**Cr**	0.19	−0.02	−0.03	**0.34**	1.00																										
	**Cu**	**0.49**	0.01	0.12	**−0.52**	−0.02	1.00																									
	**Fe**	**0.40**	0.09	**0.37**	0.08	**0.27**	−0.04	1.00																								
	**Hg**	−0.12	0.09	0.20	0.12	0.10	**−0.27**	0.12	1.00																							
	**Li**	**0.63**	0.03	0.13	**−0.53**	−0.06	**0.32**	**0.41**	−0.09	1.00																						
	**Mn**	−0.01	−0.08	−0.14	**0.65**	**0.46**	−0.14	0.25	0.05	**−0.43**	1.00																					
	**Mo**	−0.16	0.04	−0.19	**0.32**	−0.02	−0.20	0.12	0.13	−0.05	0.25	1.00																				
	**Ni**	0.11	0.05	0.08	**0.43**	**0.31**	−0.15	0.22	0.15	−0.23	**0.56**	0.10	1.00																			
	**Pb**	**0.42**	0.18	−0.17	**−0.51**	**−0.27**	**0.45**	−0.12	−0.17	**0.38**	**−0.37**	**−0.26**	−0.20	1.00																		
	**Sb**	−0.09	−0.06	0.07	**0.32**	0.08	−0.24	**0.38**	−0.10	−0.02	0.25	0.13	0.03	−0.17	1.00																	
	**Se**	0.19	0.10	−0.15	**−0.36**	−0.16	**0.29**	−0.20	−0.04	0.13	−0.13	−0.15	−0.16	**0.34**	0.01	1.00																
	**Zn**	0.13	0.01	**0.65**	−0.15	0.04	0.12	**0.65**	0.06	**0.40**	−0.20	−0.13	−0.07	0.00	**0.26**	−0.22	1.00															
**Extractable**	**Al**	0.02	0.18	−0.16	0.14	0.11	0.05	−0.13	0.15	−0.24	**0.37**	−0.01	**0.33**	0.02	−0.06	0.17	−0.21	1.00														
	**As**	0.17	**0.57**	0.14	0.07	0.11	−0.13	**0.33**	−0.01	0.08	0.20	−0.07	0.16	−0.12	**0.27**	−0.11	0.09	0.21	1.00													
	**Cd**	−0.07	0.13	0.10	−0.19	−0.07	0.00	−0.04	0.17	−0.02	0.02	0.00	0.02	−0.02	0.05	**0.31**	0.10	**0.60**	0.13	1.00												
	**Co**	0.16	0.11	0.04	**0.46**	**0.38**	0.24	0.10	0.22	0.23	**0.32**	0.16	0.24	0.20	0.08	0.13	0.02	0.25	0.20	0.06	1.00											
	**Cu**	0.07	0.21	0.00	0.07	0.04	0.12	0.06	0.13	**−0.26**	**0.27**	0.04	0.21	0.01	0.12	0.20	0.04	**0.90**	**0.27**	**0.68**	0.26	1.00										
	**Fe**	0.04	**0.33**	0.04	−0.02	−0.02	0.07	0.09	0.15	−0.09	0.22	0.05	0.20	0.06	0.10	0.17	0.04	**0.83**	**0.37**	**0.70**	**0.30**	**0.89**	1.00									
	**Hg**	0.11	0.21	0.11	0.06	0.23	0.03	0.22	0.05	0.07	0.15	−0.08	0.14	−0.08	0.15	0.00	0.07	0.17	**0.36**	0.19	0.19	0.20	0.20	1.00								
	**Li**	−0.13	−0.13	−0.13	−0.01	0.01	0.04	0.11	0.00	0.12	0.05	−0.12	−0.11	−0.11	0.08	**0.31**	0.24	**0.27**	−0.08	**0.30**	**−0.27**	**0.30**	0.23	−0.03	1.00							
	**Mn**	0.03	0.09	−0.16	0.21	0.21	0.10	−0.15	0.09	**−0.31**	**0.53**	0.00	**0.42**	−0.08	−0.03	0.18	**−0.30**	**0.91**	0.17	**0.48**	0.22	**0.81**	**0.70**	0.18	**0.26**	1.00						
	**Mo**	0.11	0.09	−0.10	−0.23	−0.14	0.19	−0.22	0.00	0.11	−0.13	0.09	−0.14	0.20	0.06	0.12	−0.19	0.17	0.12	0.15	−0.05	0.13	0.19	−0.03	−0.04	0.12	1.00					
	**Ni**	0.03	0.25	0.17	0.14	0.05	0.00	0.03	0.04	0.20	**0.40**	0.07	**0.39**	0.05	0.10	0.24	0.13	**0.88**	0.24	**0.59**	**0.31**	**0.82**	**0.82**	0.20	**0.28**	**0.84**	0.15	1.00				
	**Pb**	0.19	0.13	−0.05	−0.22	−0.21	0.19	0.03	−0.06	0.21	**−0.30**	−0.15	−0.12	**0.61**	−0.22	0.17	0.14	−0.19	−0.15	−0.23	0.00	−0.19	−0.18	−0.05	−0.07	**−0.26**	−0.09	−0.15	1.00			
	**Sb**	−0.05	0.00	0.11	**0.32**	0.06	−0.11	0.23	0.03	−0.16	**0.33**	0.09	0.24	−0.13	**0.55**	0.01	0.15	0.25	0.20	0.08	**0.32**	**0.34**	**0.29**	0.22	0.08	**0.26**	0.09	**0.33**	−0.16	1.00		
	**Se**	0.00	0.17	−0.23	0.07	0.07	0.04	−0.06	−0.05	−0.06	0.19	0.22	−0.09	0.00	0.20	0.43	−0.17	0.23	0.18	0.21	−0.02	**0.26**	0.24	−0.05	0.24	**0.30**	0.14	**0.26**	−0.14	0.07	1.00	
	**Zn**	−0.02	0.12	**0.42**	−0.10	0.07	0.15	0.19	0.07	−0.03	0.08	−0.10	0.12	−0.11	0.13	0.04	**0.40**	**0.53**	0.17	**0.62**	0.14	**0.68**	**0.70**	0.18	**0.31**	**0.52**	0.07	**0.54**	−0.21	**0.37**	0.09	1.00

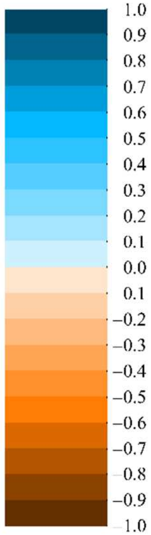

Figure S1. The Spearman's correlation matrix of studied elements for total content and extractable content (without Cr, details in text). Statistically significant coefficients (p<0.05) are bolded and marked by asterisks.
